# Identifying people at risk of rheumatoid arthritis in primary care: a qualitative study

**DOI:** 10.3399/BJGP.2024.0590

**Published:** 2025-07-01

**Authors:** Anna M Anderson, Suzanne H Richards, Caroline Flurey, Heidi J Siddle

**Affiliations:** 1 Leeds Institute of Health Sciences, University of Leeds, Leeds, UK; 2 Health and Social Sciences, University of the West of England, Bristol, UK; 3 Leeds Institute of Rheumatic and Musculoskeletal Medicine, University of Leeds, Leeds, UK

**Keywords:** arthritis, rheumatoid, early diagnosis, general practice, primary health care, qualitative research

## Abstract

**Background:**

Identification of rheumatoid arthritis (RA) in primary care is challenging and often delayed. Anticyclic citrullinated peptide (anti-CCP) antibody testing of people presenting to primary care with new-onset musculoskeletal symptoms without synovitis could help address this; those testing positive are at increased risk of developing RA.

**Aim:**

To explore how primary care clinicians currently identify and refer patients with suspected RA, and the behaviours required to implement a prediction model for guiding targeted anti-CCP testing for non-specific musculoskeletal symptoms in primary care.

**Design and setting:**

A qualitative descriptive study in primary care in England.

**Method:**

Eight GPs and eight musculoskeletal First Contact Practitioners (physiotherapists) participated in semi-structured interviews to explore their experiences of identifying and/or referring patients with suspected RA, and their views of a implementation package for the anti-CCP prediction model. Data were analysed using framework analysis.

**Results:**

Variations in practice were evident across the pathway for identifying and/or referring patients with suspected RA, including in access to and use of the anti-CCP test. Implementing the anti-CCP prediction model would require clinicians to believe its benefits outweigh its risks, engagement of primary and secondary care teams, and incorporation of the prediction model within an easily accessible and useable clinical-decision support system. Participants’ views about implementing the anti-CCP prediction model varied but were mostly positive overall.

**Conclusion:**

Implementing a prediction model to guide targeted anti-CCP testing in primary care could be feasible. Further research is required to explore the potential benefits, risks, and costs of a pathway for identifying and/or managing people at risk of RA

## How this fits in

Early identification and treatment of rheumatoid arthritis (RA) is key to improving long-term patient outcomes. Unfortunately, delays in identifying RA in primary care are common. Targeting anticyclic citrullinated peptide (anti-CCP) antibody testing could help address this by identifying people at risk of RA among those presenting with non-specific musculoskeletal symptoms in primary care. This qualitative study highlighted that primary care clinicians’ access to and use of the anti-CCP test varies, and suggests that an easily accessible clinical-decision support system for targeting anti-CCP testing could support their clinical reasoning for anti-CCP testing in people with non-specific musculoskeletal symptoms.

## Introduction

Rheumatoid arthritis (RA) is a chronic autoimmune inflammatory condition that can cause irreversible joint damage and multiple extra-articular problems.^1^ The impact of RA on patients’ health-related quality of life is profound.^2^ Direct and indirect costs are high^3^. Early initiation of disease-modifying therapies substantially improves long-term patient outcomes but is hampered by diagnostic delays.^4,5^ Patients subsequently diagnosed with RA or unclassified arthritis visit their GP a mean of four times before being referred to a rheumatologist.^5^ RA is challenging to identify in primary care as it is a rare condition with similar initial symptoms to those of many other more common conditions.^6–8^

Anticyclic citrullinated peptide (anti-CCP) antibodies, a type of anticitrullinated protein antibody, are more specific for RA than rheumatoid factor and can be detected years before clinical RA onset.^9,10^ Patients presenting to primary care with new-onset musculoskeletal (MSK) symptoms without synovitis who are anti-CCP positive can be classed as ‘at risk’ of RA as around a third develop RA within a year.^11–14^ These patients do not meet the rheumatology referral criteria in the current National Institute for Health and Care Excellence (NICE) guideline for RA in adults (NG100),^15^ which recommends referring patients with ‘suspected persistent synovitis’. However, referring at-risk individuals to rheumatology services could offer benefits by enabling them to receive monitoring, diagnosis, and early treatment initiation if required.^12^ Additionally, emerging evidence suggests that administering disease-modifying therapies to at-risk individuals could delay or prevent RA onset.^16–18^ Lifestyle interventions may also be helpful and more acceptable to patients than medications.^19^

Overuse of blood tests in primary care can have negative effects such as increasing healthcare costs and patient anxiety.^20,21^ Any changes in anti-CCP testing must therefore be evidence based and appropriately targeted.^22^ Our research group has developed a prediction model to guide targeted anti-CCP testing in primary care.^14^ Although the anti-CCP prediction model could support clinicians to identify people at risk of RA, implementing prediction models in clinical practice is challenging and rarely achieved.^23^ Even if a prediction model is embedded in a clinical decision support system (CDSS), clinicians may not use the CDSS because of factors such as mistrust, usability issues, and alert fatigue.^24,25^ Involving clinicians in developing CDSSs is vital to help address these challenges.^24,26,27^

This study was the first phase of a project focused on developing an implementation package, including a CDSS, for the anti-CCP prediction model.^14^ In line with relevant guidance,^28–32^ key intervention development steps included understanding the intervention context and the behaviours it intends to change. Previous research has provided some insights into the identification and referral of patients with suspected RA.^6–8^ However, there are many unknowns about this process in the current English primary care context. For example, previous relevant studies have not included MSK First Contact Practitioners (FCPs), (typically with a physiotherapy background) Yet the FCP workforce has recently expanded rapidly, with estimates suggesting that FCPs could see up to 50% of patients presenting to primary care with MSK conditions.^33^ Additionally, no previous studies have explored key considerations related to the anti-CCP prediction model, such as primary care clinicians’ views about identifying people at risk of RA and how these might have an impact on their behaviours.

To address these gaps, this study explored how primary care clinicians currently identify and refer patients with suspected RA, and the behaviours required to implement the anti-CCP prediction model in primary care. This is a key first step in understanding what may need to change for the anti-CCP prediction model to be successfully implemented in practice.

## Method

This qualitative descriptive study drew on the behaviour change wheel (BCW), a systematic eight-step approach for designing behaviour change interventions that involves selecting target behaviours for the intervention to change.^34^ Based on discussions within the research team, four target behaviours were identified (Box 1). The target behaviours are actions required by primary care clinicians to support implementation of the anti-CCP prediction model in primary care and associated patient management. The behaviours encompass the whole pathway from a patient presenting in primary care with new-onset MSK symptoms without synovitis through to their potential referral to rheumatology services.

**Box 1.** Target behaviours related to implementing the anti-CCP prediction model in primary care

Target behaviour
Using a CDSS incorporating the anti-CCP prediction model with appropriate patients
Organising for patients to have an anti-CCP test when supported by the CDSS
Referring patients with a positive anti-CCP test to rheumatology services
Having appropriate discussions about anything related to RA, anti-CCP testing, or rheumatology referrals with patients

Anti-CCP = anticyclic citrullinated peptide. CDSS = clinical decision support system. RA = rheumatoid arthritis.

A proposed CDSS incorporating the anti-CCP prediction model was developed as a score chart (Table 1) for completion with patients presenting to primary care with new-onset MSK symptoms without synovitis.^14^ The scores are summated to obtain a total score. The patient is referred for anti-CCP testing if the total score is equal to or greater than a score threshold. Eight- and 11-point thresholds were considered, which would lead to anti-CCP testing of approximately 40% or 19% of UK patients with new-onset MSK symptoms, respectively . Of the patients who are anti-CCP positive who are tested based on the 8- and 11-point thresholds, approximately 25% and 32% would develop RA or another inflammatory arthritis within a year, respectively.^14^ Supplementary Table S1 provides further details of the prediction model properties.

**Table 1.** Proposed CDSS incorporating the anti-CCP prediction model^14^

Predictor variables	Score
Joint pain back	–3
Joint pain neck	−2
Joint pain knee	–1
Joint pain wrist	4
Sex (male)	3
First-degree relative with RA	3
Joint pain foot/toes	3
Joint pain hand/fingers	3
Joint pain shoulder	3
Smoking history (ever)	2
Joint pain thumb	1
Total score	

Scores to be added up to obtain one total score, on a scale of –6 to 22 (for bilateral joints, pain in one or both sides should be considered an affirmative response.

Anti-CCP = anticyclic citrullinated peptide. CDSS = clinical decision support system. RA = rheumatoid arthritis.

### Patient and public involvement and project advisory group

The grant application for this study was underpinned by a focus group and email correspondence with the NICE Leeds Biomedical Research Centre patient and public involvement (PPI) group. This highlighted the importance of identifying RA earlier. Two of the PPI group members subsequently reviewed this study’s protocol. A project advisory group (PAG) involving PPI representatives, primary care clinicians, rheumatologists, and additional professionals oversaw the study and helped develop the intervention. The PAG met five times: once before this study’s commencement, once during the data-collection phase, and three times afterwards. In addition to their main PAG roles, two PAG PPI representatives helped develop a plain English summary of the results and one co-presented a public engagement video.

### Participant selection and recruitment

This study was conducted in primary care in England. GPs and FCPs (excluding trainees) were eligible if they were undertaking regular weekly clinics for a provider of NHS primary care services, which included assessing people presenting with new-onset MSK symptoms without synovitis. To obtain diverse perspectives, maximum variation purposive sampling was employed based on years of experience, organisation type(s), anti-CCP requesting practices, and experience of recruiting participants to a previous primary care anti-CCP testing study.^12^

Based on guidance for qualitative studies within larger projects,^35^ a sample size of 10–20 was prespecified and the final sample size was guided by the aim of obtaining sufficient data to provide rich insights addressing the study aim. Recruitment advertisements were shared via professional networks, including on social media. Additionally, the study details were shared directly with FCPs via an NHS community trust and private FCP provider.

### Data collection

Professional characteristics data were self-reported via email or telephone during screening. Following completion of an electronic consent form, each participant joined a single one-to-one semi-structured interview. Most interviews were held via videoconferencing. Participants who lived and/or worked in West Yorkshire were offered an in-person interview if preferred.

The first author conducted the interviews between July and October 2023 using a topic guide (Supplementary Information S1) and recorded fieldnotes during and/or after each interview. The topic guide was drafted based on the study aim, published literature, the BCW,^30^ the identified target behaviours, research team discussions, and feedback from a GP external to the study team. It was then pilot tested with a PAG FCP representative, leading to minor wording and structural changes. The finalised topic guide was piloted with a second GP external to the study team.

The first part of each interview explored participants’ experiences of identifying patients with suspected RA and referring them to rheumatology services. The interviewer then provided a brief overview of the potential implementation package for the anti-CCP prediction model. This included sharing the proposed CDSS (Table 1) and prediction model properties (Supplementary Table S1). The second part of each interview explored participants’ perspectives of the potential implementation package, including how their perspectives may influence the target behaviours. All interviews were audio- and/or video-recorded. Participants were offered a £60 recognition payment.

### Data analysis

The interviews were transcribed verbatim and pseudo-anonymised by an independent transcription company, then verified by the interviewer. NVivo software (Release 1.6, then NVivo 14), Microsoft Excel, and Microsoft Word were used for data management. Data analysis was conducted concurrently with data collection to enable issues identified in earlier interviews to inform subsequent interviews.

The framework method with a mixed deductive and inductive approach was employed to ensure the analysis was theoretically informed and novel insights were thoroughly explored.^36–38^ The deductive codes and/or categories were based on the topic guide, the BCW,^30^ and the identified target behaviours.The first four transcripts were coded independently by the first author and the senior author, who then agreed on a working analytical framework. Subsequent transcripts were indexed using the analytical framework by the first author or the senior author, who charted the data into framework matrices and developed a provisional interpretation of the data, including inductively developed themes. The interpretation was refined based on discussions with the wider study team and PAG. A behavioural analysis of the data using the BCW was also undertaken to inform the subsequent intervention development work and is reported elsewhere.

The first author is a physiotherapist working as a research fellow and the senior author is a clinical academic podiatrist working in secondary care rheumatology clinics. The wider study team brought primary care expertise. Both analysts kept reflexive journals to record reflections on aspects such as how their clinical backgrounds may have influenced the data collection and/or analysis.

## Results

Twenty-two individuals contacted the study team to express an interest in participating. Five did not respond after receiving further study information and one was excluded as they were a trainee. The remaining 16 individuals were interviewed. The interviews ranged from 34 to 87 min (median 51 min). Two were held in person. Table 2 presents the participants’ characteristics.

**Table 2.** Characteristics of participants (*n* = 16)

Characteristic^a^	Participants, *N* (%)
**Professional role**	
GP	8 (50)
FCP with a physiotherapy background	8 (50)
**Years’ experience in current role**	
<5	6 (38)
5 to <15	4 (25)
≥15	6 (38)
**Type(s) of organisation^b^**	
General practice	8 (50)
GP confederation	1 (6)
NHS community trust	5 (31)
FCP private provider	3 (19)
**Able to request the anti-CCP test**	
Yes directly	10 (63)
Yes via another professional	6 (38)
**Requested an anti-CCP blood test in the past 12 months**	
Yes	14 (88)
No	2 (13)
**Experience of recruiting participants to a previous primary care anti-CCP testing study** ^12^	
Yes	5 (31)
No	11 (69)
**Integrated care system area where workplace is located**	
Bath and North East Somerset, Swindon, and Wiltshire	1 (6)
Buckingham, Oxfordshire, and Berkshire West	3 (19)
Herefordshire and Worcestershire	1 (6)
Norfolk and Waveney	1 (6)
North East and North Cumbria	1 (6)
Suffolk and North East Essex	2 (13)
West Yorkshire	7 (44)
**Recruitment approach**	
NHS community trust	5 (31)
FCP private provider	3 (19)
Advertisement shared via professional network	5 (31)
Word of mouth	3 (19)

a Characteristics are only reported for roles in which clinicians undertake regular weekly clinics for a provider of NHS primary care services, which include assessing people with new-onset musculoskeletal symptoms without synovitis. ^b^Participants could report more than one option. Anti-CCP = anticyclic citrullinated peptide. FCP = first-contact practitioner.

### Themes overview

**Figure 1.**
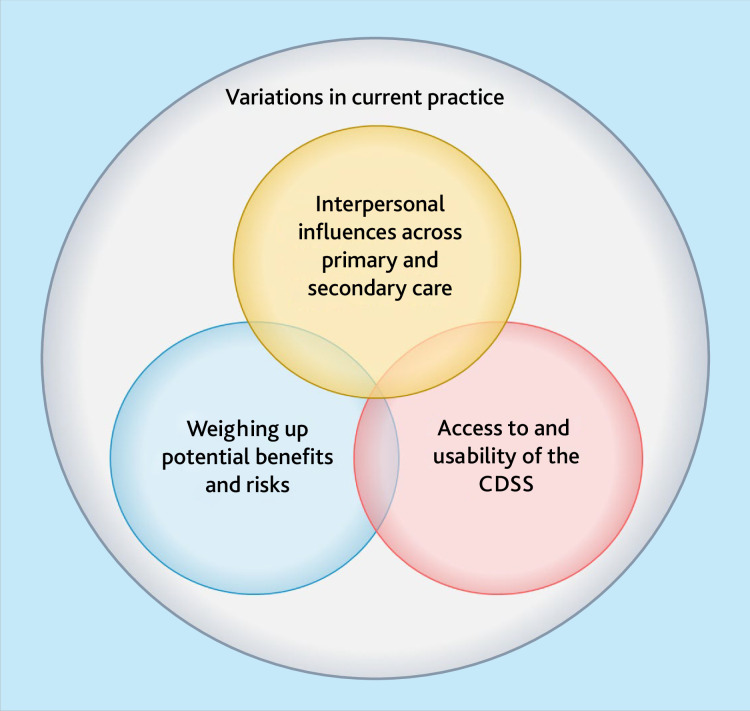
Overview of the inductively developed themes. Supplementary Information S2 provides an image description. CDSS = clinical decision support system.

One overarching theme and three main themes were developed, each representing a key area of influence on the target behaviours related to implementing the anti-CCP prediction model in primary care (Figure 1).

Considerations from all the themes influenced participants’ overall views of implementing the anti-CCP prediction model in primary care. Two GPs expressed mainly negative views, while most participants’ overall views were balanced and largely positive:

*‘I think it has potential to be useful. I think there are probably some things to think through in terms of the execution of it.’* (FCP-7)

### Overarching theme: variations in current practice

Variations in practice were evident across all stages of the process for identifying and referring patients with suspected RA including regarding use of guidelines, screening tools, and investigations; approaches to patient follow-up; and rheumatology referral procedures. These variations interlinked with the three main themes as described below. Access to and use of the anti-CCP test was a key area of variation. Some GPs reported being able to request the anti-CCP test without restrictions. Conversely, one stated the *‘*official line’ was to only request the anti-CCP test when making a rheumatology referral, and two reported that anti-CCP test requests should technically be authorised by a rheumatologist. One of the latter participants also highlighted:

*‘It’s on the naughty page in terms of blood tests* […]*.’* (GP-5)

Most FCPs indicated they need to ask a GP to request the anti-CCP test, so it was ultimately the GP’s decision about whether to request the test. However, two FCPs reported being able to request it directly in one or all the practices they work in. Three GPs and two FCPs had experience of recruiting participants to a previous anti-CCP testing study in primary care.^12^ Two felt this experience had not affected their use of the anti-CCP test, whereas the other three perceived it had led them to request it more frequently:

*‘Yeah, it’s still ordered as standard* [after the study completion]*.’* (FCP-8)

Participants’ views of their local rheumatology referral processes varied. Some described them as quick and easy, whereas others reported they were time consuming or difficult. A couple of participants highlighted potentially unnecessary steps in their local referral processes that increased the risk of errors and/or missed referrals. These steps included needing to wait for blood test and X-ray results before making a referral, secretaries needing to send referrals, and referrals being sent for triage:

‘*Send for triage then involves someone else getting involved, which mostly happens but very occasionally, doesn’t happen.’* (GP-8)

Variations in participants’ caseloads were also relevant. Although both GPs and FCPs highlighted the proposed CDSS could be used with a large volume of patients, its adoption would have more of an impact on FCPs because of their purely MSK caseload:

*‘That is probably going to be a bit difficult because if I’m to ask every single patient about every single thing on there* […]*.’ (FCP-3)*

### Main theme: weighing up potential benefits and risks

Participants’ beliefs about potential benefits and risks related to the anti-CCP prediction model had a strong influence on their willingness to engage in the target behaviours. Most participants felt that identifying people at risk of RA could improve patient outcomes but would also pose risks. A key concern was that labelling patients as at risk of RA may cause them unnecessary fear, worry, or anxiety. It was suggested that this could lead to patients becoming ‘quite regular service users’ and have a substantial impact on their lives:

*‘*[…] *they don’t even know if they’re going to get it but they’re waiting for that day for it to happen, and that can actually limit their function, almost like a self-fulfilling prophecy sort of thing, that they’re going to go down a route of pain and discomfort and things like that.’*

(FCP-2)

Patient education was considered key to minimising this risk, although there was concern about whether clinicians would have time to provide sufficient education. Some participants suggested easily accessible patient education resources would be helpful. Conversely, a minority of participants felt that too much information could be alarming or overwhelming. Participants generally believed providing lifestyle advice to at-risk individuals would be appropriate. However, most had concerns about biological medications intended to delay and/or prevent RA onset, with a few reporting they would want evidence about their benefits and risks:

*‘I guess, it would be a bit of number needed to treat, ’cause you’ve still got quite a large chunk of those patients who aren’t going to progress.’* (GP-2)

Many participants felt the proposed CDSS could support clinical reasoning, with a few commenting that the predictor variables and/or prediction model looked appropriate (Table 2, Supplementary Table S1). Despite this, a minority of participants queried whether using the CDSS would change their practice and all participants raised some concerns about the predictor variables, prediction model, and/or supporting evidence base. Thes included the focus on anti-CCP positivity rather than RA development being confusing and less clinically relevant:

*‘The outcome you’re really interested in is who gets rheumatoid arthritis and as that kind of middleman it muddies the waters a bit about, oh, you know, are they CCP positive or negative?’* (GP-7)

Some of the differences in participants’ beliefs about potential benefits and risks of the anti-CCP prediction model appeared to relate to variations in their practice, including how frequently they request the anti-CCP test. For example, some participants felt using the CDSS would fit in with their current practice:

*‘I don’t think it would be too onerous, I think it would be acceptable, especially in line of what we’re already doing, I think it would be reasonable.*’ (GP-4 whose local processes recommend routinely requesting the anti-CCP test at the same time as rheumatoid factor)

Others had concerns about the cost and/or workload implications of using the CDSS, including that it would lead to a substantial increase in anti-CCP testing:

*‘*[…] *why do I want to go round testing* forty *per cent or* nineteen *per cent of everybody who presents with musculoskeletal symptoms with an anti-CCP? Because you’re now asking me to do an enormous number of blood tests.’* (GP-6, whose local processes recommend only requesting anti-CCP when making a rheumatology referral)

Correspondingly, many but not all participants felt the 11-point threshold would be preferable to the 8-point threshold as that would lead to anti-CCP testing of 19% rather 40% of people with new-onset MSK symptoms (Supplementary Table S1). Although participants generally felt individuals who are anti-CCP positive should be referred to rheumatology services, some were concerned about overburdening rheumatology services and increasing waiting times:

*‘I’m thinking more from how services are, sort of, under pressure, and I think unnecessarily increasing the waiting times* […]*.’* (FCP-6)

### Main theme: interpersonal influences across primary and secondary care

Interpersonal influences across primary and secondary care appeared to influence all the target behaviours. Some FCPs reported that they did not usually ask GPs to request specific blood tests, with one suggesting:

*‘GPs are better placed to know what bloods to order’.* (FCP-1)

Conversely, a few FCPs reported they sometimes specify which tests to request:

*‘I think if we’re the ones that have assessed the patient and we’re the ones requesting, I think it does make sense to be specific about what we’re asking.’* (FCP-4)

Variations in practice appeared to be linked to interpersonal influences across primary and secondary care. For example, a couple of FCPs reported that their approach to organising anti-CCP tests varied with the general practice they are working in. This appeared to relate at least partly to whether the GPs trust FCPs to act autonomously. One of these participants highlighted the importance of GPs supporting FCPs’ use of the proposed CDSS:

*‘I think the main thing for us from an FCP point of view is making sure* […] *GPs have the equal education because, you know, some of them are, like, oh, these FCPs have gone crazy ordering all these blood tests* […]*.’* (FCP-2)

Positive relationships, experiences, and feedback from the rheumatology team appeared to be a facilitator to making rheumatology referrals, while concern about referrals being rejected was a barrier. A couple of participants highlighted that referrals of patients at risk of RA (without clinical RA symptoms) would probably be rejected at present. Correspondingly, a few participants highlighted that rheumatology teams would need to be on board with primary care clinicians referring patients at risk of RA to them:

*‘*[…] *if rheumatology were really on board with that and it was a directive that they were … it was something that they were keen to kind of explore, then obviously that would be a great thing to then start and kind of move patients that way.’* (FCP-5)

Factors related to patients also appeared to be important. For example, participants discussed their knowledge and skills related to communicating effectively and/or involving patients in decision making, although some reported that they do not usually involve patients in decisions about requesting the anti-CCP test specifically. This appeared to be related to perceptions of normal practice, time constraints, and assumptions that patients will want blood tests:

*‘*[…] *there may be a prejudice on my part that I assume patients do want investigation, if it’s on offer.’* (GP-1)

### Main theme: access to and usability of the CDSS

Nearly all the participants emphasised the importance of ensuring that the proposed CDSS is quick and easy to access and use. One GP stated that he would like ‘a nice laminated copy’, whereas most participants felt a version integrated within electronic health record (EHR) systems would be ideal. A few participants highlighted that they would like to access the CDSS on a separate website. Participants’ preferences appeared to be linked to variations in their practice, particularly regarding their use and views of EHR systems. For example, one FCP explained his use of integrated versus web-based tools depends on which EHR system he is using:

*‘We’ve got one on a* [System X] *template which works perfectly. The* [System Y] *one’s a load of garbage so I just use the one on the website, it’s dead quick.’* (FCP-8)

Most participants stated they would prefer to complete the CDSS themselves rather than providing a patient questionnaire and liked the suggested layout:

*‘It’s nice and simple, it’s quite clear. All it needs is some tick boxes and an automatic calculation at the bottom and that would be perfect really.’* (FCP-1)

A few participants felt it would be helpful to provide further guidance about how to assess the predictor variables (Table 1), for example, by specifying how long the joint pain needs to have been present. There also appeared to be a need for further guidance on which patients to use the CDSS with. Areas of confusion included whether the CDSS should be used with patients with suspected inflammatory arthritis, and whether its use should be limited to patients without a clear cause for their symptoms:

*‘I guess it’s those patients where you’ve, sort of, done your assessment and you can’t really figure out, you know, what’s going on with them from a mechanical element.’* (FCP-7)

A few participants felt clinicians may forget to use the proposed CDSS. Correspondingly, multiple participants felt it would be helpful to get into a habit of using the CDSS and/or have prompts to use it. Some participants felt a pop-up would be helpful, but a few identified feasibility concerns or felt they would ignore a pop-up:

*‘Because my instinct with any pop-up is to get rid of it* […]*.’* (GP-4)

## Discussion

### Summary

This study has provided novel insights into how clinicians identify and refer patients with suspected RA in the current English primary care context and identified considerations for implementing a prediction model to guide targeted anti-CCP testing in primary care. Numerous variations in practice were evident, some of which related to the differing roles of GPs and FCPs. Variations between individuals from the same professional group were also apparent. Successful implementation of the anti-CCP prediction model is likely to require clinicians to believe its benefits outweigh its risks, engagement from primary and secondary care teams, and incorporation of the prediction model within an easily accessible and useable CDSS. Although participants’ overall views of implementing the anti-CCP prediction model varied, most felt it could potentially be helpful.

Implementing the anti-CCP prediction model in primary care is one step in a potential pathway for identifying and monitoring and/or managing people at risk of RA. Recent studies have developed scoring systems for stratifying patients who are anti-CCP positive into groups based on their likelihood of developing inflammatory arthritis,^12,13^ and suggested disease-modifying therapies could delay or prevent RA onset in at-risk individuals.^16–18^ Future research is required to address remaining uncertainties about the feasibility, risks, and benefits of identifying and monitoring and/or managing people at risk of RA, including risks known to be important to patients and clinicians.^19,22^ Considering implementation early is key to ensuring that new interventions have the potential to make a positive difference in the real world;^28^ therefore, this study provides an important foundation for future research to build on.

### Strengths and limitations

This study comprehensively explored considerations for implementing an anti-CCP prediction model in primary care, including by identifying barriers and facilitators to four target behaviours. While the findings are likely to be valuable for future research, the immediate applicability of some findings to clinical practice may be limited as organisations such as NICE do not currently provide guidance on anti-CCP testing in patients with non-specific MSK symptoms without synovitis or managing individuals who are anti-CCP positive at risk of RA.^15^ This study included GPs and FCPs, two key stakeholder groups likely to be involved in identifying patients at risk of RA.

The heterogeneous group of participants with wide geographical places of work was a strength of this study. However, there may have been some bias in the recruitment as it relied on clinicians responding to advertisements or details about the study shared via an NHS community trust and private FCP provider. Given that the primary care workforce is diversifying, other primary care clinicians, such as advanced nurse practitioners, may also have a role in this area.

The involvement of PPI representatives was a key strength. This ensured that the study addressed an issue of importance to patients, the design was appropriate from a patient perspective, and the findings were shared in accessible and engaging formats. Any changes in anti-CCP testing in primary care must address diverse patients’ needs and will require collaboration with secondary care. Although PPI representatives and rheumatologists were actively involved in the PAG, they were not included as participants. Future research is therefore required to build on this study by exploring the views of a wider range of patients and rheumatologists in depth. Another limitation was that participants knew the interviewer was involved in developing an implementation package for the anti-CCP prediction model, which may have made them reluctant to criticise it.

### Comparison with existing literature

Previous studies have highlighted variations in how GPs identify and refer patients with suspected RA, including regarding their use of and perspectives on the anti-CCP test.^6,7^ This study suggests differing local availability of the anti-CCP test and being involved in anti-CCP research studies may affect clinicians’ use of the anti-CCP test. The importance of good interpersonal relationships between GPs and rheumatology teams has also been noted previously, with some GPs reporting difficulty accessing rheumatology services.^6–8^ The present study adds to this by highlighting that engagement from rheumatology teams is likely to be key to implementing the anti-CCP prediction model. This study also provides novel insights into the interpersonal relationships between GPs and FCPs, with GPs’ trust in FCPs appearing to influence the approach of some FCPs to organising anti-CCP tests. FCPs’ beliefs about whether it is appropriate to ask GPs to request specific blood tests varied, aligning with research highlighting uncertainty about the boundaries of the FCP role.^39^

As with this study, previous research has highlighted the importance of optimising access to and usability of CDSSs.^24,26,40^ Integration within EHRs is generally considered the best way to optimise access and is recommended by the NHS England CDSS implementation guide.^27^ In contrast, this study demonstrates that some clinicians may prefer to access CDSSs in other formats; hence, providing CDSSs in more than one format may be valuable.

Based on their qualitative study, Ford *et al* ^24^ made recommendations to consider when developing a new primary care CDSS, including providing visual aids for communicating the findings with patients. While some participants in this study felt that a patient education resource would be valuable, others raised concerns about providing too much information. Additionally, some participants reported they do not usually involve patients in decisions about requesting the anti-CCP test specifically. This corresponds with previous research highlighting limited shared-decision and information provision by GPs when ordering blood tests.^41,42^

### Implications for research and practice

This study identified numerous considerations for developing an implementation package for the anti-CCP prediction model in primary care, which have been taken forward to the subsequent intervention development work. Future research that includes a range of stakeholders will be required to evaluate the feasibility and potential clinical- and cost-effectiveness of implementing the anti-CCP prediction model. That could potentially be embedded within a broader evaluation of a pathway integrating the anti-CCP prediction model alongside a risk-stratifying system for individuals who are anti-CCP positive.^12,13^ This study also highlights the need for further research in areas such as how to monitor and/or manage patients at risk of RA, and how clinicians should communicate information about anti-CCP testing to patients.

Variations across all stages of the process for identifying and/or referring patients with suspected RA were highlighted in this study. Addressing some variations may be helpful. For example, rheumatology referral procedures in certain areas could be streamlined to reduce the work burden on clinicians and limit the risk of errors and/or missed referrals.

## Data

The study data include in-depth information about participants’ experiences and perspectives; therefore, it is not available to help ensure that participants’ identities remain confidential.
